# Navigating the blurred boundary: Neuropathologic changes versus clinical symptoms in Alzheimer’s disease, and its consequences for research in genetics

**DOI:** 10.1177/13872877251317543

**Published:** 2025-02-16

**Authors:** Catarina Xavier, Nádia Pinto

**Affiliations:** 1 i3S — Instituto de Investigação e Inovação em Saúde, Universidade do Porto, Porto, Portugal; 2 IPATIMUP — Instituto de Patologia e Imunologia Molecular da Universidade do Porto, Porto, Portugal; 3 CMUP — Centro de Matemática da Universidade do Porto, Porto, Portugal

**Keywords:** Alzheimer's disease, biomarkers, diagnosis, genome-wide association studies, imaging

## Abstract

During decades scientists tried to unveil the genetic architecture of Alzheimer's disease (AD), recurring to increasingly larger sample numbers for genome-wide association studies (GWAS) in hope for higher statistical gains. Here, a retrospective look on the most prominent GWAS was performed, focusing on the quality of the diagnosis associated with the used data and databases. Different methods for AD diagnosis (or absence) carry different levels of accuracy and certainty applied to both subsets of cases and controls. Furthermore, the different phenotypes included in these databases were explored, as several incorporate other ageing comorbidities and might be encompassing many confounding agents as well. Age of the samples’ donors and origin populations were also investigated as these could be biasing factors in posterior analyses. A tendency for looser diagnostic methods in more recent GWAS was observed, where greater datasets of individuals are analyzed, which may have been hampering the discovery of associated genetic variants. Specifically for AD, a diagnostic method conveying a clinical outcome may be distinct from the disease neuropathological assessment, since the first has a practical perspective that not necessarily needs a confirmation. Due to its properties and complex diagnosis, this work highlights the importance of the neuropathological confirmation of AD (or its absence) in the subjects considered for research purposes to avoid reaching statistically weak and/or misleading conclusions that may trigger further studies with powerless groundwork.

## Introduction

Dementia places an enormous burden, not only on patients, but also on their caregivers, families, and society at large. It is expected that people living with dementia will rise from 55 M in 2019 to 139 M in 2050.^1^ Alzheimer's disease (AD) is the most common cause of dementia and is rapidly becoming one of the most lethal and burdensome diseases of this century, both economically and socially.^2^ Despite the massive effort from worldwide scientific community (e.g., the National Institutes of Health invested more than $56B in the last 15 years^3^), there is still no treatment to cure dementia.

Beyond AD, other neurodegenerative diseases can lead to dementia, and although the different causes are associated with different symptom patterns and brain abnormalities, specific diagnosis is still challenging. Clinicians tend to use worldwide developed guidelines to establish normative diagnosis, and in AD two of these are widely applied: the NINCDS-ARDRDA and the Diagnostic and Statistical Manual of Mental Disorders (DSM). The NINCDS-ADRDA were put forward as a joint effort by the National Institute of Neurological and Communicative Disorders and Stroke and the Alzheimer's Disease and Related Disorders Association and focus on the diagnosis of AD considering four stages of AD diagnostic certainty (definite, probable, possible, and unlikely).^4^ On the other hand, the DSM is a series of guidelines that are published by the American Psychiatric Association that focus on the classification of mental disorders and can also be applied to the diagnosis of dementia or cognitively compromised individuals. Although, these criteria are applied for the clinical diagnosis of the disease, more recent diagnostic guidelines integrated biomarker data for probable and possible AD for research purposes.^5^ It is noteworthy that the latest diagnostic criteria for AD were recently published and include the use of biomarkers for its biological definition.^6^ Indeed, the distinction between neuropathologic changes and clinical symptoms may become blurred, as AD terminology is often used to refer to either prototypical clinical syndromes without neuropathologic verification of AD changes. These entities may overlap to some degree but should not be confused, as a syndrome is not an etiology, but rather a clinical consequence of one or more diseases. The difficulty in distinguishing the two entities may have had also consequences in scientific research setting, as we will discuss later.

Brain changes associated with AD that define it as a unique neurodegenerative disease among the various disorders that can lead to dementia are the deposition of amyloid-β (Aβ) plaques due to deficient processing of Aβ peptide and the formation of neurofibrillary tangles composed mainly of abnormally phosphorylated tau (p-tau) protein.^7^ The three best-validated neuroimaging biomarkers for AD are medial temporal lobe atrophy on magnetic resonance imaging (MRI) and posterior cingulate and temporoparietal hypometabolism on fluorodeoxyglucose (FDG) - positron emission tomography (PET), respectively; as measures of neurodegeneration, and cortical Aβ deposition on amyloid-PET imaging. Aβ, p-tau, and neurodegeneration can also be determined by cerebrospinal fluid (CSF) biomarkers, with extensive research efforts currently directed towards the development of serum and plasma biomarkers. Several automated platforms have been developed for the analysis of Aβ_1–42_, p-tau 181, and total tau.^8–10^

Heritability plays an important role in AD,^11^ studies involving twins showed that the risk of developing AD is ∼60–80% dependent on heritable factors.^12^ The discovery of AD-associated genes may provide crucial insights for the biological understanding of the disease, and links between risk variant genetics and AD pathophysiology were already established for amyloidogenic pathway, modulation of the immune response, lipid dysfunction, cholesterol, endocytosis, vascular factors, among others – see for example.^13–24^ Additionally, the presence (or absence) of risk alleles in an individual's genome might allow the assessment of his/her risk of developing the disease.

From the genetic point of view, two subtypes of the disease are generally considered: familial AD, and sporadic AD (∼20 times more prevalent than the first), since the genetic contributions are known to be different in one and another case. The first is characterized by an earlier onset, while the second typically appears in sporadic cases after 65 years old,^25^ which has been supporting the designation of late-onset AD (LOAD). The latter designation may however be avoided since it is prone to confusion as sporadic, non-familial, AD may also be diagnosed in younger subjects. The heritability of familial AD is typically explained by the occurrence of rare variants in a few genes, including *APP, PSEN1*, and *PSEN2*, with a strong effect; whereas sporadic AD has been associated with common variants, that are expected to have a small influence when analyzed individually, but a high impact when studied together.^26–28^ In any case, allele ε4 of the *APOE* gene has been reported as the strongest genetic risk for the disease since the early 1990s.^29^ *APOE* allele ε3 is the most common worldwide despite the allele ε4 being described as the ancestral allele.^30^ Alleles ε4 and ε2 showed a variable distribution worldwide and even within Europe, the frequencies of the strongest risk factor for AD—ε4— ranging between 0.045 for some countries in Asia to 0.407 for some others in Africa.^29–31^ Specifically, *APOE* allele ε4 has been associated with an estimated 3- to 4-fold increased risk of sporadic AD, whereas the other more than 70 risk alleles identified (which may be only a small fraction of the linked variants^32^) are presented as being associated with much smaller contributions.^33^

The traditional approach for identifying medically actionable population-based information relies in the development of case-control association studies, under which the difference in allele frequency between cases and controls is used to estimate the causal effect that a particular variant may have on the disease or phenotype of interest, taking into account all potential confounders. As a great number of variants are expected to be encountered linked to sporadic AD, genotypic associations have been sought through case-control genome-wide association studies (GWAS). GWAS face the challenge of ultrahigh dimensionality due to the bulk of genetic markers analyzed, which has been expected to be counterbalanced with the analysis of huge datasets of individuals.

International consortia and databases containing genomic data from individuals considering their sporadic AD status have been used by the scientific community in research settings, specifically in GWAS. Focused on AD-related case-control GWAS using data from these international consortia, this work was triggered by the fact that when considering such a complex phenotype as AD, the level of confidence of the status of the subjects differ between the used databases, as the stringency of the criteria for inclusion of subjects varies widely. A recent review sheds light on the caveats of increasing the sample size of some GWAS disregarding the quality of the data that have been feeding the statistical models.^34^ The review discusses the decreasing explained heritability in the found loci despite the increased number of samples and pinpoints the decrease of clinically verified cases and increased number of cases-by-proxy. Furthermore, it also identifies a lack of independency among studies as more recent studies englobe the samples from earlier papers, among other issues.^34^

Indeed, beyond the difficulties transversal to all the phenotypes, sporadic AD is a remarkably complex disease to study under a case-control framework due to its specificities, which may blur both cases and controls subsets. Examples of these specificities rely 1) on being an age-related condition with a very long preclinical stage, which may lead to the erroneous inclusion of subjects in the control group that are not manifesting the disease but already at a preclinical stage of the disease, and 2) on the difficulty of specific diagnosis, which may lead that patients with other forms of dementia are considered in the subset of cases. Worse, in some of the late studies, subjects whose AD status is established by proxy, i.e., not established directly but considering the one of the family members, have been included^33,35,36^ which increases even more the blurring between and within both cases and controls subsets. The immediate consequence of this poor assessment of the AD status of the subjects is that statistical inferences regarding correlation between specific genetic variants and the disease are inaccurate,^34^ which may bring to light genetic variants falsely correlated, and camouflage others truly associated with the disease.

Aware of these complexities, in this work we provide a systematic review of the sporadic AD case-control GWAS, along with a critical analysis that will provide insights on the strengths and weaknesses of the scientific developments achieved so far. Additionally, we will focus on the standards accomplished by the international databases containing AD-related genomic information that have been used in GWAS, and we will focus on the subjects considered and results obtained by a set of 40 GWAS. Finally, we discuss the results and present the concluding remarks, providing a general overview of the state of the art of the latest AD GWAS.

### International databases containing genomic information of AD cases and/or controls used in GWAS

A total of 25 consortia gathering genomic data from both cases and controls were used in GWAS where the phenotype of interest is AD—for more details see Supplemental Table 1. These consortia gathered data from 120 genomic databases, 15 and 1 of which having simultaneously contributed for two and three of them, respectively, increasing the risk of profiles’ repetition. In Table 1, a summary of the data contained in this set of 120 worldwide genomic databases (herein, databases) is presented, based on publicly available information. These databases were gathered according to their characteristics, including the number of sampling times the individuals were subjected to, i.e., cross-sectional (non-recurrent sampling: one subject, one sampling time) and longitudinal databases (recurrent sampling: one subject, several samplings over time – despite genomic information being mostly stable over time, other types of data are collected). Cross-sectional databases were divided into phenotype-oriented studies (n = 42, including others than AD) and others (n = 7, including national biobanks, brain banks and commercial DNA databases). Longitudinal databases were divided into cohort (target populations, n = 58) and panel studies (no target populations, n = 5). For a total of eight databases (n = 8) no information was publicly available.

**Table 1.** Summary data concerning 112 out of the 120 genomic databases used in the set of 40 AD case-control GWAS, considering the information publicly available. For more details, see Supplemental Table 1. NA: not applicable. No information was found for 8 databases.

		Cross sectional		Longitudinal	
		Phenotype oriented (n = 42)	Others (Commercial DNA databases, national biobanks and brain banks, n = 7)	Panel study (Without a specific target population, n = 5)	Cohort (With a specific target population, n = 58)
Phenotype of interest	Alzheimer's disease (AD)	28	NA	NA	12
	AD and other dementias	4	NA	NA	3
	AD, dementia and cognitive impairment	4	NA	NA	17
	Stroke and dementia	1	NA	NA	-
	Other neurologic diseases including AD	1	NA	NA	-
	Other neurologic diseases not including AD	3	NA	NA	2
	Cardiovascular diseases	1	NA	NA	3
	Others	-	NA	NA	21
Group of interest	Phenotype only	42	-	-	37
	Older populations (age-based design)	-	-	-	21
	General population	-	7	5	-
Data Collection	Unrelated	36	7	5	54
	Familial	6	-	-	4
Base Population	USA (minorities)	28 (2)	3	3	26 (6)
	Europe	9	3	2	24
	Latin America	5	-	-	2
	Asia	-	-	-	5
	Australia	-	-	-	1
	Worldwide	-	1	-	-
Minimum age (cases)	<60	1	1	1	6
	[60–70]	14	1	-	16
	≥70	-	-	-	5
	NA	27	5	4	31
Minimum age (controls)	<60	1	1	1	8
	[60–70]	12	1	-	11
	[70–80]	-	-	-	6
	≥80	1	-	-	5
	NA	28	5	4	28

AD is the phenotype targeted in some databases (n = 40, 33.3%), the number increasing when considering other dementias beyond AD and/or cognitive impairments (n = 68, 56.7%). Additionally, databases based in other phenotypes are considered for AD-GWAS, such as strokes or cardiovascular diseases (n = 5, 4.2%), considering the genomic information of these subjects for the subsets of either cases or controls. Although the inclusion of more diverse databases increases undoubtedly the number of available samples, which is normally the limiting factor identified with GWAS, it will also increase the number of confounding factors that will bring noise to the statistical interpretation of the overall results. Groups of interest (specific groups of people that were sampled despite presenting or not a specific phenotype, e.g., “older individuals”) were also explored, particularly for cohort databases since the remaining are either focused on a specific phenotype of interest or do not target a specific group of individuals (general population). The cohort databases targeted either older individuals (n = 21, 17.5%), or specific phenotypes (n = 37, 67.7%).

Another major question that can be posed is if the data that have been gathered for these several GWAS is representative of the worldwide genetic pool. We explored the base populations sampled in all databases and observed that mostly European or European descendants are targeted (n = 98, 81.7%). Other emerging base populations include US minority populations (mostly of African-American descent), Asian, and Latin America countries (Table 1). Since there is a differential frequency of risk alleles in different populations (e.g., *APOE* ε4 allele, as previously mentioned), the ancestry of the subjects should be considered to more accurately perceive insights into disease associations and risk factors.

Additionally, in some databases, cases and controls by proxy (i.e., subjects whose AD status is not evaluated directly, but inferred considering the one of the family members) are also considered, which increases even more the confounding effects. Ten databases use this familial (and inferring) approach for sampling, four of which are longitudinal and six phenotype-oriented. Little information is made publicly available on how the sampling procedure took place within the families, but in some cases criticizable and conflicting procedures are known. For example, in some databases it is stated that all the members of families with history of sporadic AD in at least two of them are collected as patients without further investigation; inversely, another database mentioned that unaffected siblings of AD subjects were considered as controls. This procedure implies that healthy individuals may be included in the subset of patients, while individuals with genetics of risk, eventually in the preclinical stage of the disease, may be included in the subset of controls. Although the mere presence of an AD-affected person in the family is not deterministic that other relatives will develop the disease, the genetic constellation of these people has a higher probability of sharing risk alleles. The idea of sampling by proxy is very tempting and often used to increase the sample size; however, the risk of introducing unwanted genetic contributions (either by increasing the AD-set with AD-free samples or increasing the number of controls with AD-genetics) will generate a blurred picture of the genetics of the disease.

Unquestionably, the accuracy of the statistical inferences relies on the correct status of the subjects, both cases and controls, that is particularly tricky to establish in a disease like AD, specifically under a genomics framework that is mostly stable during the subjects’ lifetime. Indeed, if a clear description of AD's genetic landscape is sought after, then a clear-cut differentiation between AD patients and others (controls) needs to be obtained. The clinical assessment of AD can be extremely challenging and thus when utilizing the genomic information of AD cases without biomarker's confirmation, individuals with other types of dementia are likely to be included in the subset of cases. Conversely, individuals in the preclinical stage of the disease may be included as controls and considered through the statistical analyses as “protected” genomes.

The assessment of the AD status of the subjects was categorized as mention to either NINDCS-ADRDA or DSM III-IV criteria was done, or otherwise (i.e., no reference to any guidelines or criteria). Within each one of these categories, six subcategories were considered depending on the methods used for establishing the AD status: 1) Autopsy for all – i.e., databases that mention having autopsy data for all the subjects, 2) Imaging for all – i.e., imaging data (e.g., MRI, PET) for all, 3) CSF for all – cerebrospinal fluid biomarkers data for, 4) Mixed biomarkers’ analysis for all – i.e., databases that have either autopsy, imaging or CSF data for all, 5) Unspecified or diverse methods for the different subjects – i.e., databases that present: a) unspecific diagnostic methods (e.g., only clinical interviews), b) neuropsychological tests (e.g., Mini-Mental State Examination), c) mixed diagnostic methods (e.g., autopsy for part of the set and neuropsychological tests for the remaining), 6) Non-clinical interview, self- or third party-assessment – i.e., databases that base the establishment of the AD status of the subjects on self- or third-party-declarations. Additionally, a seventh category was added since for a few databases no publicly available information was found regarding the methodology used for establishing AD status of the subjects.

In what concerns the subset of cases, a total of 44 databases describe following the NINCDS-ADRDA criteria (44/112, 39.2%), not specifying however either possible/probable or probable/definite AD status (Table 2). These guidelines describe the usage of different diagnostic methods to achieve a specific AD status, for example the definite AD status can only be reached using either autopsy confirmation or histopathological data. However, probable and possible AD status are more ambiguous and based on clinical observation and neuropsychological tests. In addition to NINCDS-ADRDA, only four (4/44, 9.1%) databases describe neuroimaging data for the entire dataset and one (1/44, 2.3%) database has mixed biomarkers’ analysis for the entire dataset. Out of these 44, 39 (39/44, 88.6%) databases use unspecific and/or diverse methods for AD assessment (not specified as either possible/probable or probable/definite). A total of 56 databases (56/112, 50%) do not describe following either NINCDS-ADRDA or DSM III/IV criteria, however 14 databases present either autopsy (6/56, 10.7%), imaging (6/56, 10.7%) or mixed biomarkers’ analysis for the entire dataset (2/56, 3.6%). Similarly to the previous category, the majority of databases describe the use of unspecific and/or diverse methods for the entire or parts of the dataset (40/56, 71.4%) with one database basing their AD diagnostic in a non-clinical interview/self- or third party-assessment (1/56, 1.8%). Finally, 12 (12/112, 10.7%) databases did not share any information on AD-assessment. This indicates that different levels of confidence on the AD diagnosis of cases exist not only for subjects from different databases, but also for those included in the same database.

**Table 2.** Summary data concerning AD and AD-free assessment methods for the 120 genomic databases used in the set of the 40 GWAS. For a total of eight databases no information was publicly available. For more details, see Supplemental Table 1.

AD status	Criteria	Assessment methods	Cross sectional		Longitudinal	
			Phenotype oriented	Others	Panel study	Cohort
			Case-control studies (n = 42)	Commercial DNA databases, national biobanks and brain banks (n = 7)	Without a specific target population (n = 5)	With a specific target population (n = 58)
AD-assessment (Cases)	NINCDS-ADRA	Autopsy for the complete set of samples	-	-	-	-
		Imaging for the complete set of samples	1	-	-	3
		CSF for the complete set of samples	-	-	-	-
		Mixed biomarkers’ analysis for the complete set of samples	-	-	-	1
		Unspecified methods or diverse for the different subjects (including absence of biomarkers’ analyses)	23	1	1	14
	DSM III or IV	Imaging for the complete set of samples	-	-	-	1
		Unspecified methods or diverse for the different subjects (some with only neuropsychological tests or clinical interviews as minimum criteria)	1	-	-	-
	Without reference	Autopsy for the complete set of samples	3	3	-	-
		Imaging for the complete set of samples	-	-	-	6
		CSF for the complete set of samples	-	-	-	-
		Mixed biomarkers’ analysis for the complete set of samples	-	-	-	2
		Unspecified methods or diverse for the different subjects (some with only neuropsychological tests or clinical interviews as minimum criteria)	13	1	4	22
		Non-clinical interview, self- or third party -assessment	-	1	-	-
	No information available		1	1	-	9
AD-free assessment (Controls)	NINCDS-ADRA	Autopsy for the complete set of samples	-	-	-	-
		Imaging for the complete set of samples	1	-	-	2
		CSF for the complete set of samples	-	-	-	-
		Mixed biomarkers’ analysis for the complete set of samples	-	-	-	1
		Unspecified methods or diverse for the different subjects (including absence of biomarkers’ analyses)	6	-	-	5
	DSM III or IV	Imaging for the complete set of samples	-	-	-	-
		Unspecified methods or diverse for the different subjects (some with only neuropsychological tests or clinical interviews as minimum criteria)	1	-	-	1
	Without reference	Autopsy for the complete set of samples	3	3	-	-
		Imaging for the complete set of samples	-	-	-	9
		CSF for the complete set of samples	-	-	-	-
		Mixed biomarkers’ analysis for the complete set of samples	-	-	-	-
		Unspecified methods or diverse for the different subjects (some with only neuropsychological tests or clinical interviews as minimum criteria)	25	-	5	23
		Non-clinical interview, self- or third party -assessment	1	4	-	5
	No information available		5	-	-	12

Equally or even more complex is the identification of subjects to be part of the subset of AD controls (Table 2). Considering that AD is a common and very heterogeneous disease, with different degrees of severity and patterns of evolution, the introduction of controls based on limited diagnostic methods could represent adding individuals that have not manifested the disease at the time of sampling but that will develop it later in life. Since AD is an age-related disease, this risk is even greater when using young individuals as subject controls. For this subset of subjects, 15 (15/112, 13.4%) databases declared using NINCDS-ADRDA criteria, and from these three describe imaging data for the entire dataset (3/15, 20%) and one describe mixed biomarkers’ analysis for the entire dataset (1/15, 6.7%). The remaining databases describe unspecific or diverse methods for the different subjects (11/15, 73.3%). Most databases do not declare following a single and specific criteria for control assessment (78/112, 69.6%). From these, most describe unspecified and/or diverse methods (53/78, 67.9%), followed by non clinical /self- or third-party assessment (9/78, 11.5%). Few databases refer either imaging (10/78, 12.8%) or autopsy data (6/78, 7.7%) for the entire set of controls. Finally, it is noteworthy that for seventeen databases (17/112, 15.1%) no publicly available information was found regarding the diagnostic methods used for the recruitment of controls.

Impressively, approximately half of the considered databases do not present any information on age minimum requirements (if any) for either AD-case (67/112, 59.8%), or control subjects (65/112, 58.0%). Among the databases disclosing information on the age of the subjects, most starts in the range 60–70 years old (31/112, 27.7% Table 1) for the subset of cases. When considering the subset of controls or subjects by proxy, minimum age presents more diverse intervals ranging from as early as 20 and 35 years old to >80, presenting the most common interval 60–70 years old as for cases (24/112, 21.4%).

Finally, for a total of 8 databases no descriptive information was found about sampling methods and design. As discussed previously, several databases do not share information on established age thresholds for AD and control samples or do not further explain the methodologies used to establish the AD status of the individuals, jeopardizing the inference of accuracy of the results and the reliability of the AD status of the subjects (Supplemental Table 1).

### Genome wide association studies

In this section, we revise 40 AD case-control GWAS that, since 2007, built their subjects’ datasets using the international databases mentioned above, and reported at least one new risk loci to those previously discovered^20,33,35–72^ (Supplemental Table 2). A special focus will be devoted to the cohorts used in each case, analyzing the criteria considered for establishing the subjects’ status (both AD and AD-free).

**Figure 1.**
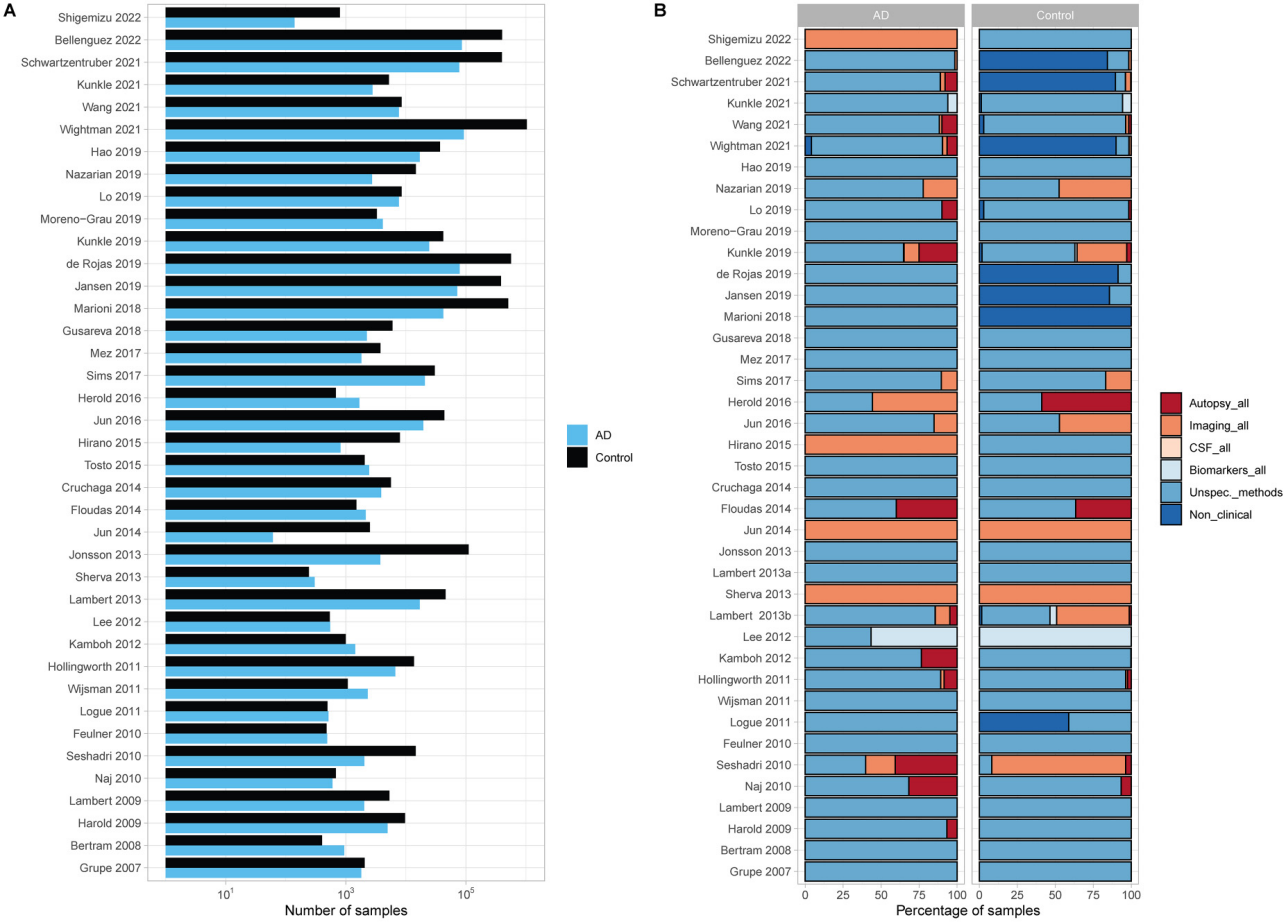
(A) TOTAL number of subjects with AD (cases) and ad-free (controls) status, for each GWAS analyzed. (B) Percentage of samples that are inserted in databases designed using either imaging, autopsy, CSF mixed biomarker analysis, unspecific or several diagnostic methods, or non-clinical and self-/third-party-assessment for AD diagnosis and control AD-free assessment, for the complete set of subjects, for each GWAS.

Following the theoretical expectation that increasing the number of subjects may be the key to discover markers associated to AD, GWAS have been showing a clear trend to increase the number of analyzed subjects, the most recent including subjects whose AD status was established by proxy (Figure 1A). Indeed, in some cases an impressive number of subjects was analyzed, as is the case of^36^ where ∼1.127 M of subjects were considered (∼44k cases, ∼47k cases by proxy, ∼718k controls and ∼318k controls by proxy). Aiming for sample increase, the more recent studies englobed the earlier ones as previously discussed in a recent review that discusses the lack of independency among recent GWAS,^34^ to which we include yet another case: Lo et al. and Wang et al.^67,70^

As previously mentioned, different target phenotypes were allowed to join in these studies (such as other dementias and mild cognitive impairment but also subjects with other diseases or conditions—see Table 1), as well as databases not designed for clinical purposes (such as the commercial DNA database 23andMe, https://www.23andme.com/) that are currently used to find controls (mainly, but also cases). Here, we analyze the composition of the cohorts of subjects considered for the mentioned GWAS, considering the methods for AD and AD-free assessment mentioned in the previous section (Figure 1B, Supplemental Tables 3 and 4).

When considering all samples used for the considered GWAS (Table 3, Figure 1B), the AD assessment method for the majority of the case-subjects analyzed fall in the broader category of unspecific/diverse methods (92.2%), with no information on the proportion of subjects in the different categories regarding the accuracy of the diagnosis. For a small proportion of subjects either autopsy (3.9%), imaging data (3.2%) or a mixed of different biomarkers’ analyses (0.1%) were analyzed for the whole AD dataset. Finally, AD subjects with non-clinical interview/self- third-party-assessment (0.6%) were also considered. Interestingly, we observe a decrease in the percentage of samples coming from databases with imaging for the whole dataset from older to more recent GWAS; however, this trend is inverted for autopsy (but less expressive). Indeed, 5 out of the 40 GWAS (12.5%) present 50% of their sample composition with imaging data, four of these GWAS are within the timespan of 2007–2018 (14.8%). Seventeen GWAS (42.5%) presented 100% of sample composition with AD assessment of unspecific/diverse methods and 33 (82.5%) presented 50%. Of these, 13 (48.1%) were older GWAS (2007–2018) and 4 (30.8%) GWAS from 2019 onwards. Figure 1B represents visually these trends, where a clear decrease in the proportion of samples with imaging for whole the dataset is visible for more recent GWAS, while the bigger proportions of this category show up in GWAS from 2013–2016.

**Table 3.** Methods for AD-assessment of subjects of both cases and controls subsets, for the analyzed genome-wide association studies (all the GWAS, n = 40; older GWAS 2007–2018, n = 27 and recent GWAS from 2019 onwards, n = 13) and number of GWAS presenting above 50%, 0% and 100% of samples from databases with a specific AD/control assessment.

		AD CASES						CONTROLS					
		Imaging for all	Autopsy for all	CSF for all	Biomarkers for all (diverse)	Unspec. / diverse methods*	Non clinical interview	Imaging for all	Autopsy for all	CSF for all	Biomarkers for all (diverse)	Unspec. / diverse methods*	Non clinical interview
Samples (N, all GWAS)		19,224	23,849	-	700	562,326	3807	119,035	6445	-	4540	615,231	3E + 06
Samples (%, all GWAS)		3.2	3.9	-	0.1	92.2	0.6	3.2	0.2	-	0.1	16.5	80.0
Samples (N, GWAS 2007–2018)		9431	3936	-	311	131,263	-	63,631	2500	-	2561	246,614	507,955
Samples (%, GWAS 2007–2018)		6.5	2.7	-	0.2	90.6	-	7.7	0.3	0.0	0.3	30.0	61.7
Samples (N, GWAS 2019–2022)		9793	19,913	-	389	431,063	3807	55,404	3945	-	1979	368,617	2E + 06
Samples (%, GWAS 2019–2022)		2.1	4.3	-	0.1	92.7	0.8	1.9	0.1	-	0.1	12.7	85.1
GWAS (N, all)	>50%	5	-	-	1	33	-	3	1	-	1	27	7
	0%	24	28	40	35	4	39	27	28	40	34	3	28
	100%	4	-	-	-	17	-	2	-	-	-	16	1
GWAS (N, 2007–2018)	>50%	4	-	-	1	21	-	3	1	-	1	19	2
	0%	18	20	27	26	3	27	20	20	27	25	3	24
	100%	3	-	-	-	13	-	2	-	-	-	13	1
GWAS (N, 2019–2022)	>50%	1	-	-	-	12	-	-	-	-	-	8	5
	0%	6	8	13	9	1	12	7	8	13	9	-	4
	100%	1	-	-	-	4	-	-	-	-	-	3	-

*Unspecific / diverse methods.

Control, or AD-free, assessment was also analyzed for the entire batch of 40 GWAS and was based on the same criteria and categories as mentioned earlier. Table 3 and Figure 1B showcase a descriptive summary of the overall sample composition and the sample composition per GWAS, respectively. Concerning a general picture of the controls, most samples originate from databases that base their AD-free diagnosis on a non-clinical interview /self- or third-party-assessment (80%), followed by unspecific/diverse methods (16.5%). Free AD status based on imaging data for whole dataset was only obtained for few subjects (3.2%). The remaining categories presented only residual representations. Interestingly, more control samples are coming from databases that use non-clinical or self-/third-party assessment for AD diagnosis, 7 GWAS present more than 50% of their sample composition from these databases (17.5%), 5 of these are recent GWAS (38.5%). Furthermore, we observe a decrease in the number of GWAS using either imaging or autopsy data for more than 50% of their sample composition (3 to 0 GWAS and 1 to 0 GWAS from old to recent timespan for imaging and autopsy, respectively).

Despite achieving a higher number of subjects, unfortunately the more recent GWAS are not reflecting the increase in the SNPs’ significance. On this regard, it should be mentioned that the statistical significance of the results is differently reported by the different works, which hampers the direct comparison of the obtained results in the different studies. For example, odd ratios comparing the odds of the events (phenotype of interest or its absence) on the carriers and non-carriers of the variants of interest have been the standard way to quantify and present the strength of the observations in case-control studies. Nevertheless, even this standard measure is neither presented nor information allowing its computation is provided in 9 out of the 40 analyzed papers. In Supplemental Figure 1A is showed the odds ratio (OR) interval found for the set of 31 GWAS that provided this information, ordered by the corresponding median. Noticeably, twelve GWAS presented maximum OR above 2.5, eight of them are related to *APOE* SNPs or SNPs in linkage disequilibrium with *APOE* (more details in Supplemental Tables 5 and 6), which may enclose redundancy of the results. Interestingly, for the remaining four GWAS, a relatively low number of samples was used for the discovery phase (5609–115,040). Two studies with high OR values were performed using controls with ages > 70 years old^54^ and > 85 years old with intact cognition capacities.^20^ These results reinforce the hypothesis that a more careful selection of AD cases (better and uniform diagnosis methods) and controls (older ages and a good assessment of AD absence) aids in the recognition of AD's genetic signals. Furthermore, the remaining two studies with the highest OR values were performed in African American populations.^60,71^

Supplemental Figure 1B depicts the mean and median OR values plotted against the total number of samples used for the discovery phase in the 31 analyzed GWAS that provided these statistics. Indeed, GWAS reaching close to half a million to one million samples, displayed maximum ORs of 1.06–1.31, clearly contradicting the general tendency of sampling increase to gain statistical power (Supplemental Table 5). Correlation tests were calculated between the total N and the mean and median OR, but were found statistically non-significant. At some extent this result could be explained if more variants with smaller ORs associated had been identified by studies with larger datasets. Nevertheless, for the set of 31 GWAS for which OR results were provided, only a residual positive correlation (R^2 ^= 0.001) between the number of analyzed subjects and the number of identified associated variants was detected.

**Figure 2.**
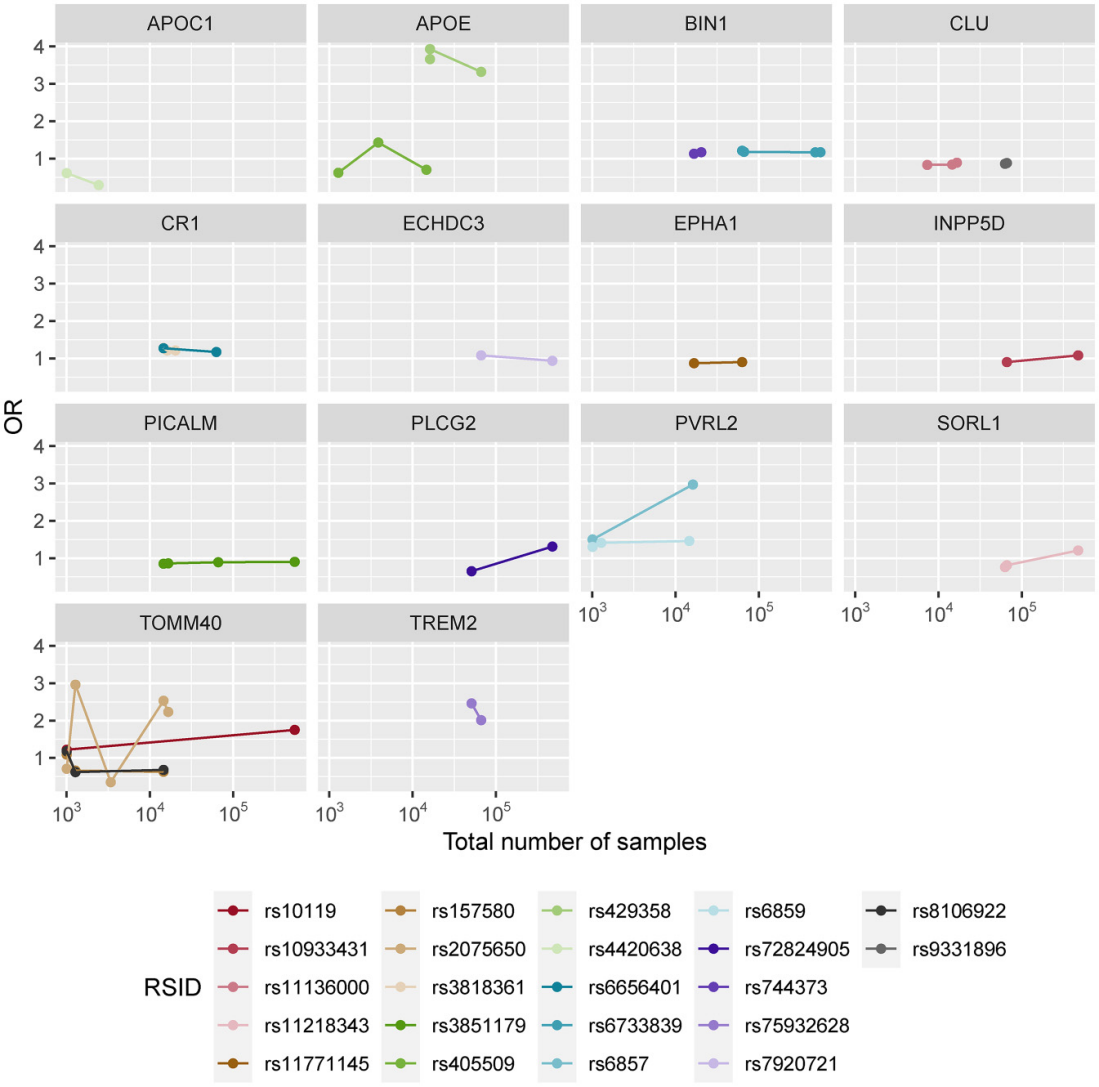
Marker specific odds ratios per the total number of samples for the set of 22 overlapping SNPs found in at least two GWAS, separated by genes. In each case the number of points represent the number of GWAS that identified the corresponding marker as AD-associated.

**Figure 3.**
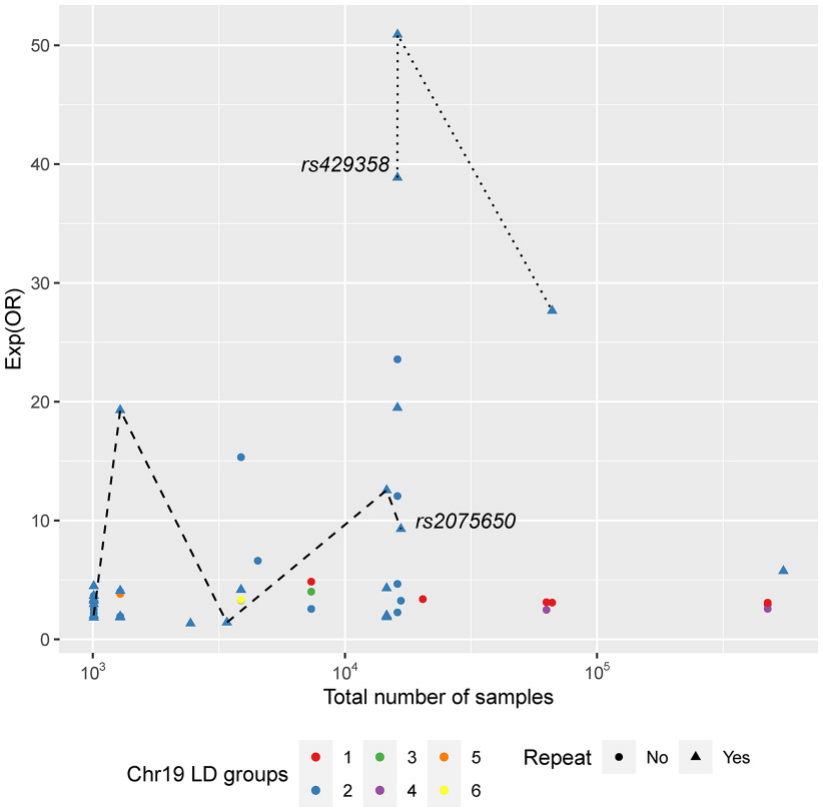
Marker specific odds ratios (exp scale) per total number of samples for all the Chromosome 19 variants found to be AD-associated in at least one of the analyzed GWAS. LD groups were established considering LDmatrix output (Supplemental Figure 2) and two variants are represented by the same color if and only if they belong to the same LD group. Point shapes consider if the SNP was found in more than one GWAS (repeat, triangle) or otherwise (circle). Two SNPs are highlighted as examples.

Aiming for a better understanding of the OR variation between studies, all SNPs found to be associated with the disease in their different GWAS were analyzed both individually and in a per-chromosome basis (Figures 2 and 3, Supplemental Figures 2–4, Supplemental Table 7). Overall, a set of 22 SNPs was found to overlap in at least two GWAS and corresponding ORs were explored individually (Figure 2). Additionally, as some markers were observed in close physical locations in the chromosome, linkage disequilibrium (LD) calculations were performed for all SNPs within the same chromosome using LDmatrix^73^ (Supplemental Figure 4) to assess the OR variation of linked markers. A per-chromosome analysis concerning LD was carried out for all the identified variants, and a special attention was devoted to those cases where some AD associated variants were found to be in LD (Supplemental Figure 4). Chromosome 19 showed the highest number of associated SNPs and it is represented as an example in Figure 3, where an absence of correlation between OR and number of samples (N) is visible. Indeed, when investigating specific SNPs unstable patterns of OR appear, as in the two SNP examples in Figure 3 and in several examples in Figure 2. These results corroborate the previous analyses in Supplemental Figure 1 and both reinforce the possibility that increasing datasets does not reflect *per se* statistical gains and that more deliberation on the quality of diagnosis or AD-absence assessment must be considered. Possibly, the combination of samples with different diseases with similar phenotypes and the introduction of poorly classified controls blurred the overall genetic signal of AD, preventing the clarification of the disease's genetic architecture.

## Discussion and concluding remarks

New sequencing methods have provided high throughput capacity for the analysis of an abundant amount of DNA markers (even full genomes) and a profuse number of samples. Undoubtedly, it has brought impressive scientific progress where big data is needed to solve scientific questions. However, the mishandling of these technologies and particularly of the data analysis that it involves is also increasing. The genetic constellation of AD has sparked the interest of several scientific groups along the years; however, the scientific gain during the last years, despite the amount of produced data, is still limited. This study provides a retrospective view of a collection of GWAS that provided new insights and clues on the genetic composition of sporadic AD. We try to make sense of the little statistical gain that accompanied the gigantic increase in sample size and interpret what caveats we can hopefully overcome. Indeed, we observed a lack of correlation between an increase in sample size and an increase in statistical significance of the described loci. We propose that the choice of collecting more data that satisfy basic requirements not considering the quality of diagnostic assessment will pool together different neurodegenerative diseases that translate in similar behavioral phenotypes, leading to a blurred picture of the genetics of a specific disease. Furthermore, introducing data from various databases that were designed differently brings a heterogeneity to the data that can add more confounding factors, limiting the accuracy of statistical outcomes. Indeed, a more careful selection of these databases should be taken into consideration when defining solid groups of case-control samples for research purposes. With this purpose, scientists should weigh in a uniform diagnosis of the disease and disease-free for all samples. It is, however, bizarre that we are discussing an issue that was clearly addressed 13 years ago in NINCDS-ADRDA guidelines,^5,74^ where was established the need for biomarkers’ pathological confirmation for subjects of scientific research investigations. These minimum requirements have not been transversally applied neither for the subset of cases nor for the one of controls, and it is noteworthy the apparent lack of information in some databases that prevent a knowledgeable choice of data taking into account solid diagnosis methods and AD-free assessments.

Additionally, when selecting controls, age should be taken into the equation, since AD is strongly age-correlated. Typically, subsets of cases and controls are built matching as much as possible the sex and age of the subjects, the latter lacking rational in the case of AD, at least under the framework of a standard case-control association study. Indeed, a 70 years-old subject, without any AD symptom included in the subset of controls, may be a case if he/she would be re-analyzed one year later, which of course beclouds the statistical analyses since in a first instance his/her (risk) genomic profile would be classified as AD protected. Indeed, the genomic information is (majority) the same during the lifespan of the individual, which implies that under a typical case-control study the same genomic profile may cross from the control subset (considered by the statistical model as AD protected), to the case subset (AD risk) at any point of the life of the individual. This risk is particularly high in a disease as AD that is common in the population, has a complex diagnosis and a long preclinical phase, the first symptoms appearing in old or very old individuals. It may be hypothesized that AD is inevitable as long as the individual live long enough,^75^ but if that is the premise the age of the individuals has to be considered as covariate associating the genetic variants with the age of appearing the first symptoms. This approach is not the one that has been carried out, under which protected (controls) and affected (cases) genomic profiles have been compared.

Another factor that can introduce variability and bias the results is the choice of populations from where the data is being gathered from, as AD genetics might manifest differently among populations as previously observed for the *APOE* ɛ4 allele.^29–31^ It is well known that *APOE* genotype affect the risk factor for developing the disease and therefore, should be taken into equation when recalculating risk factors of the remaining markers. Ideally, parallel research in different biogeographic populations should take place to minimize the possibility of risk allele dilution, whilst still capturing worldwide diversity.

Furthermore, the more recent GWAS utilize data that originate from the same databases and thus, one might question the independency of their results (e.g.,^36,65,67,70^). Also noteworthy is that statistical analyses are not standardized, and provided data frequently not allow the computation of alternative statistics. This makes the comparison between papers difficult and blocks testing the assay for reproducibility.

AD genetics is a very complex issue that will continue to awe researchers worldwide as we carry on with this challenge that might have multiple answers. Instead of bulging sample numbers irrespectively of data quality, we hypothesize that lower sample sizes but with highly curated data would provide more fruitful insights regarding the true genetic portrait of the disease. With highly curated data we mean both AD and control subjects with neuropathological verification from worldwide populations. Indeed, the boundary between neuropathologic changes and clinical symptoms in AD has become increasingly unclear, as its terminology may be applied to typical clinical syndromes even without neuropathologic confirmation. These concepts may partially overlap but should not be conflated; however, a syndrome is a clinical outcome resulting from one or more diseases, not the cause itself. The ongoing challenge of differentiating these two aspects may not be of an extreme importance in the clinical setting, but can tremendously impact AD-related scientific research, specifically in the search of associated genetic variants.

## Supplemental Material

sj-docx-2-alz-10.1177_13872877251317543 - Supplemental material for Navigating the blurred boundary: Neuropathologic changes versus clinical symptoms in Alzheimer’s disease, and its consequences for research in genetics Supplemental material, sj-docx-2-alz-10.1177_13872877251317543 for Navigating the blurred boundary: Neuropathologic changes versus clinical symptoms in Alzheimer’s disease, and its consequences for research in genetics by Catarina Xavier and Nádia Pinto in Journal of Alzheimer's Disease

sj-xlsx-3-alz-10.1177_13872877251317543 - Supplemental material for Navigating the blurred boundary: Neuropathologic changes versus clinical symptoms in Alzheimer’s disease, and its consequences for research in genetics Supplemental material, sj-xlsx-3-alz-10.1177_13872877251317543 for Navigating the blurred boundary: Neuropathologic changes versus clinical symptoms in Alzheimer’s disease, and its consequences for research in genetics by Catarina Xavier and Nádia Pinto in Journal of Alzheimer's Disease

## References

[1] Long S Benoist C Weidner W . *World Alzheimer Report 2023: Reducing dementia risk: never too early, never too late.* London: Alzheimer's Disease International, 2023.

[2] Alzheimer's Association. 2023 Alzheimer's disease facts and figures. Alzheimers Dement 2023; 19: 1598–1695. 36918389 10.1002/alz.13016

[3] National Institutes of Health. RePORT - Research Portfolio Online Reporting Tools, https://report.nih.gov/ (accessed June 22, 2023).

[4] McKhann G Drachman D Folstein M , et al. Clinical diagnosis of Alzheimer's disease. Neurology 1984; 34: 939–939. 6610841 10.1212/wnl.34.7.939

[5] McKhann GM Knopman DS Chertkow H , et al. The diagnosis of dementia due to Alzheimer's disease: recommendations from the national institute on aging-Alzheimer's association workgroups on diagnostic guidelines for Alzheimer's disease. Alzheimers Dement 2011; 7: 263–269. 21514250 10.1016/j.jalz.2011.03.005 PMC3312024

[6] Jack CR Jr Andrews JS Beach TG , et al. Revised criteria for diagnosis and staging of Alzheimer's disease: Alzheimer's association workgroup. Alzheimers Dement 2024; 20: 5143–5169. 38934362 10.1002/alz.13859 PMC11350039

[7] Mattson MP . Pathways towards and away from Alzheimer's disease. Nature 2004; 430: 631–639. 15295589 10.1038/nature02621 PMC3091392

[8] Blennow K Shaw LM Stomrud E , et al. Predicting clinical decline and conversion to Alzheimer's disease or dementia using novel Elecsys Aβ(1–42), pTau and tTau CSF immunoassays. Sci Rep 2019; 9: 19024. 31836810 10.1038/s41598-019-54204-z PMC6911086

[9] Hansson O Seibyl J Stomrud E , et al. CSF Biomarkers of Alzheimer's disease concord with amyloid-β PET and predict clinical progression: a study of fully automated immunoassays in BioFINDER and ADNI cohorts. Alzheimers Dement 2018; 14: 1470–1481. 29499171 10.1016/j.jalz.2018.01.010 PMC6119541

[10] Kaplow J Vandijck M Gray J , et al. Concordance of Lumipulse cerebrospinal fluid t-tau/Aβ42 ratio with amyloid PET status. Alzheimers Dement 2020; 16: 144–152. 31914216 10.1002/alz.12000 PMC7061432

[11] Consortium TB Anttila V Bulik-Sullivan B , et al. Analysis of shared heritability in common disorders of the brain. Science 2018; 360: eaap8757. 10.1126/science.aap8757 PMC6097237 29930110

[12] Gatz M Reynolds CA Fratiglioni L , et al. Role of genes and environments for explaining Alzheimer disease. Arch Gen Psychiatry 2006; 63: 168–174. 16461860 10.1001/archpsyc.63.2.168

[13] Di Marco LY Venneri A Farkas E , et al. Vascular dysfunction in the pathogenesis of Alzheimer's disease–A review of endothelium-mediated mechanisms and ensuing vicious circles. Neurobiol Dis 2015; 82: 593–606. 26311408 10.1016/j.nbd.2015.08.014

[14] Bennett RE Robbins AB Hu M , et al. Tau induces blood vessel abnormalities and angiogenesis-related gene expression in P301L transgenic mice and human Alzheimer's disease. Proc Natl Acad Sci USA 2018; 115: E1289–E1298. 10.1073/pnas.1710329115 PMC5819390 29358399

[15] Verheijen J Sleegers K . Understanding Alzheimer disease at the interface between genetics and transcriptomics. Trends Genet 2018; 34: 434–447. 29573818 10.1016/j.tig.2018.02.007

[16] Naj AC Schellenberg GD . Genomic variants, genes, and pathways of Alzheimer's disease: an overview. Am J Med Genet B Neuropsychiatr Genet 2017; 174: 5–26. 27943641 10.1002/ajmg.b.32499 PMC6179157

[17] Holstege H van der Lee SJ Hulsman M , et al. Characterization of pathogenic SORL1 genetic variants for association with Alzheimer's disease: a clinical interpretation strategy. Eur J Hum Genet 2017; 25: 973–981. 28537274 10.1038/ejhg.2017.87 PMC5567154

[18] Cuyvers E De Roeck A Van den Bossche T , et al. Mutations in ABCA7 in a Belgian cohort of Alzheimer's disease patients: a targeted resequencing study. Lancet Neurol 2015; 14: 814–822. 26141617 10.1016/S1474-4422(15)00133-7

[19] Keren-Shaul H Spinrad A Weiner A , et al. A unique microglia type associated with restricting development of Alzheimer's disease. Cell 2017; 169: 1276–1290.e1217. 28602351 10.1016/j.cell.2017.05.018

[20] Jonsson T Stefansson H Steinberg S , et al. Variant of TREM2 associated with the risk of Alzheimer's disease. N Engl J Med 2013; 368: 107–116. 23150908 10.1056/NEJMoa1211103 PMC3677583

[21] Parhizkar S Arzberger T Brendel M , et al. Loss of TREM2 function increases amyloid seeding but reduces plaque-associated ApoE. Nat Neurosci 2019; 22: 191–204. 30617257 10.1038/s41593-018-0296-9 PMC6417433

[22] Sala Frigerio C Wolfs L Fattorelli N , et al. The major risk factors for Alzheimer's disease: age, sex, and genes modulate the microglia response to aβ plaques. Cell Rep 2019; 27: 1293–1306.e1296. 31018141 10.1016/j.celrep.2019.03.099 PMC7340153

[23] Hansen DV Hanson JE Sheng M . Microglia in Alzheimer's disease. J Cell Biol 2018; 217: 459–472. 29196460 10.1083/jcb.201709069 PMC5800817

[24] Griciuc A Patel S Federico AN , et al. TREM2 Acts downstream of CD33 in modulating microglial pathology in Alzheimer's disease. Neuron 2019; 103: 820–835.e827. 31301936 10.1016/j.neuron.2019.06.010 PMC6728215

[25] Zhu XC Tan L Wang HF , et al. Rate of early onset Alzheimer's disease: a systematic review and meta-analysis. Ann Transl Med 2015; 3: 38. 25815299 10.3978/j.issn.2305-5839.2015.01.19 PMC4356853

[26] Cacace R Sleegers K Van Broeckhoven C . Molecular genetics of early-onset Alzheimer's disease revisited. Alzheimers Dement 2016; 12: 733–748. 27016693 10.1016/j.jalz.2016.01.012

[27] Goate A Chartier-Harlin MC Mullan M , et al. Segregation of a missense mutation in the amyloid precursor protein gene with familial Alzheimer's disease. Nature 1991; 349: 704–706. 1671712 10.1038/349704a0

[28] Sherrington R Rogaev EI Liang Y , et al. Cloning of a gene bearing missense mutations in early-onset familial Alzheimer's disease. Nature 1995; 375: 754–760. 7596406 10.1038/375754a0

[29] Belloy ME Napolioni V Greicius MD . A quarter century of APOE and Alzheimer's disease: progress to date and the path forward. Neuron 2019; 101: 820–838. 30844401 10.1016/j.neuron.2019.01.056 PMC6407643

[30] Fullerton SM Clark AG Weiss KM , et al. Apolipoprotein E variation at the sequence haplotype level: implications for the origin and maintenance of a major human polymorphism. Am J Hum Genet 2000; 67: 881–900. 10986041 10.1086/303070 PMC1287893

[31] Singh PP Singh M Mastana SS . APOE Distribution in world populations with new data from India and the UK. Ann Hum Biol 2006; 33: 279–308. 17092867 10.1080/03014460600594513

[32] Holland D Frei O Desikan R , et al. The genetic architecture of human complex phenotypes is modulated by linkage disequilibrium and heterozygosity. Genetics 2021; 217: iyaa046. 10.1093/genetics/iyaa046 PMC8045737 33789345

[33] Bellenguez C Küçükali F Jansen IE , et al. New insights into the genetic etiology of Alzheimer's disease and related dementias. Nat Genet 2022; 54: 412–436. 35379992 10.1038/s41588-022-01024-z PMC9005347

[34] Escott-Price V Hardy J . Genome-wide association studies for Alzheimer's disease: bigger is not always better. Brain Commun 2022; 4: fcac125. 10.1093/braincomms/fcac125 PMC9155614 35663382

[35] Schwartzentruber J Cooper S Liu JZ , et al. Genome-wide meta-analysis, fine-mapping and integrative prioritization implicate new Alzheimer's disease risk genes. Nat Genet 2021; 53: 392–402. 33589840 10.1038/s41588-020-00776-w PMC7610386

[36] Wightman DP Jansen IE Savage JE , et al. A genome-wide association study with 1,126,563 individuals identifies new risk loci for Alzheimer's disease. Nat Genet 2021; 53: 1276–1282. 34493870 10.1038/s41588-021-00921-z PMC10243600

[37] Grupe A Abraham R Li Y , et al. Evidence for novel susceptibility genes for late-onset Alzheimer's disease from a genome-wide association study of putative functional variants. Hum Mol Genet 2007; 16: 865–873. 17317784 10.1093/hmg/ddm031

[38] Bertram L Lange C Mullin K , et al. Genome-wide association analysis reveals putative Alzheimer's disease susceptibility loci in addition to APOE. Am J Hum Genet 2008; 83: 623–632. 18976728 10.1016/j.ajhg.2008.10.008 PMC2668052

[39] Harold D Abraham R Hollingworth P , et al. Genome-wide association study identifies variants at CLU and PICALM associated with Alzheimer's disease. Nat Genet 2009; 41: 1088–1093. 19734902 10.1038/ng.440 PMC2845877

[40] Lambert JC Heath S Even G , et al. Genome-wide association study identifies variants at CLU and CR1 associated with Alzheimer's disease. Nat Genet 2009; 41: 1094–1099. 19734903 10.1038/ng.439

[41] Naj AC Beecham GW Martin ER , et al. Dementia revealed: novel chromosome 6 locus for late-onset Alzheimer disease provides genetic evidence for folate-pathway abnormalities. PLoS Genet 2010; 6: e1001130. 10.1371/journal.pgen.1001130 PMC2944795 20885792

[42] Seshadri S Fitzpatrick AL Ikram MA , et al. Genome-wide analysis of genetic loci associated with Alzheimer disease. JAMA 2010; 303: 1832–1840. 20460622 10.1001/jama.2010.574 PMC2989531

[43] Feulner TM Laws SM Friedrich P , et al. Examination of the current top candidate genes for AD in a genome-wide association study. Mol Psychiatry 2010; 15: 756–766. 19125160 10.1038/mp.2008.141

[44] Logue MW Schu M Vardarajan BN , et al. A comprehensive genetic association study of Alzheimer disease in African Americans. Arch Neurol 2011; 68: 1569–1579. 22159054 10.1001/archneurol.2011.646 PMC3356921

[45] Wijsman EM Pankratz ND Choi Y , et al. Genome-wide association of familial late-onset Alzheimer's disease replicates BIN1 and CLU and nominates CUGBP2 in interaction with APOE. PLoS Genet 2011; 7: e1001308. 10.1371/journal.pgen.1001308 PMC3040659 21379329

[46] Hollingworth P Harold D Sims R , et al. Common variants at ABCA7, MS4A6A/MS4A4E, EPHA1, CD33 and CD2AP are associated with Alzheimer's disease. Nat Genet 2011; 43: 429–435. 21460840 10.1038/ng.803 PMC3084173

[47] Kamboh MI Demirci FY Wang X , et al. Genome-wide association study of Alzheimer's disease. Transl Psychiatry 2012; 2: e117. 10.1038/tp.2012.45 PMC3365264 22832961

[48] Lee JH Cheng R Barral S , et al. Identification of novel loci for Alzheimer disease and replication of CLU, PICALM, and BIN1 in Caribbean hispanic individuals. Arch Neurol 2011; 68: 320–328. 21059989 10.1001/archneurol.2010.292 PMC3268783

[49] Lambert JC Ibrahim-Verbaas CA Harold D , et al. Meta-analysis of 74,046 individuals identifies 11 new susceptibility loci for Alzheimer's disease. Nat Genet 2013; 45: 1452–1458. 24162737 10.1038/ng.2802 PMC3896259

[50] Sherva R Tripodis Y Bennett DA , et al. Genome-wide association study of the rate of cognitive decline in Alzheimer's disease. Alzheimers Dement 2014; 10: 45–52. 23535033 10.1016/j.jalz.2013.01.008 PMC3760995

[51] Lambert JC Grenier-Boley B Harold D , et al. Genome-wide haplotype association study identifies the FRMD4A gene as a risk locus for Alzheimer's disease. Mol Psychiatry 2013; 18: 461–470. 22430674 10.1038/mp.2012.14 PMC3606943

[52] Jun G Asai H Zeldich E , et al. PLXNA4 Is associated with Alzheimer disease and modulates tau phosphorylation. Ann Neurol 2014; 76: 379–392. 25043464 10.1002/ana.24219 PMC4830273

[53] Floudas CS Um N Kamboh MI , et al. Identifying genetic interactions associated with late-onset Alzheimer's disease. BioData Min 2014; 7: 35. 25649863 10.1186/s13040-014-0035-z PMC4300162

[54] Cruchaga C Karch CM Jin SC , et al. Rare coding variants in the phospholipase D3 gene confer risk for Alzheimer's disease. Nature 2014; 505: 550–554. 24336208 10.1038/nature12825 PMC4050701

[55] Tosto G Fu H Vardarajan BN , et al. F-box/LRR-repeat protein 7 is genetically associated with Alzheimer's disease. Ann Clin Transl Neurol 2015; 2: 810–820. 26339675 10.1002/acn3.223 PMC4554442

[56] Hirano A Ohara T Takahashi A , et al. A genome-wide association study of late-onset Alzheimer's disease in a Japanese population. Psychiatr Genet 2015; 25: 139–146. 26049409 10.1097/YPG.0000000000000090

[57] Jun G Ibrahim-Verbaas CA Vronskaya M , et al. A novel Alzheimer disease locus located near the gene encoding tau protein. Mol Psychiatry 2016; 21: 108–117. 25778476 10.1038/mp.2015.23 PMC4573764

[58] Herold C Hooli BV Mullin K , et al. Family-based association analyses of imputed genotypes reveal genome-wide significant association of Alzheimer's disease with OSBPL6, PTPRG, and PDCL3. Mol Psychiatry 2016; 21: 1608–1612. 26830138 10.1038/mp.2015.218 PMC4970971

[59] Sims R van der Lee SJ Naj AC , et al. Rare coding variants in PLCG2, ABI3, and TREM2 implicate microglial-mediated innate immunity in Alzheimer's disease. Nat Genet 2017; 49: 1373–1384. 28714976 10.1038/ng.3916 PMC5669039

[60] Mez J Chung J Jun G , et al. Two novel loci, COBL and SLC10A2, for Alzheimer's disease in African Americans. Alzheimers Dement 2017; 13: 119–129. 27770636 10.1016/j.jalz.2016.09.002 PMC5318231

[61] Gusareva ES Twizere JC Sleegers K , et al. Male-specific epistasis between WWC1 and TLN2 genes is associated with Alzheimer's disease. Neurobiol Aging 2018; 72: 188.e183–188.e112. 10.1016/j.neurobiolaging.2018.08.001 PMC6769421 30201328

[62] Marioni RE Harris SE Zhang Q , et al. GWAS On family history of Alzheimer's disease. Transl Psychiatry 2018; 8: 99. 29777097 10.1038/s41398-018-0150-6 PMC5959890

[63] Jansen IE Savage JE Watanabe K , et al. Genome-wide meta-analysis identifies new loci and functional pathways influencing Alzheimer's disease risk. Nat Genet 2019; 51: 404–413. 30617256 10.1038/s41588-018-0311-9 PMC6836675

[64] de Rojas I Moreno-Grau S Tesi N , et al. Common variants in Alzheimer's disease and risk stratification by polygenic risk scores. Nat Commun 2021; 12: 3417. 34099642 10.1038/s41467-021-22491-8 PMC8184987

[65] Kunkle BW Grenier-Boley B Sims R , et al. Genetic meta-analysis of diagnosed Alzheimer's disease identifies new risk loci and implicates Aβ, tau, immunity and lipid processing. Nat Genet 2019; 51: 414–430. 30820047 10.1038/s41588-019-0358-2 PMC6463297

[66] Moreno-Grau S de Rojas I Hernández I , et al. Genome-wide association analysis of dementia and its clinical endophenotypes reveal novel loci associated with Alzheimer's disease and three causality networks: the GR@ACE project. Alzheimers Dement 2019; 15: 1333–1347. 31473137 10.1016/j.jalz.2019.06.4950

[67] Lo MT Kauppi K Fan CC , et al. Identification of genetic heterogeneity of Alzheimer's disease across age. Neurobiol Aging 2019; 84: e241–243.e249. 10.1016/j.neurobiolaging.2019.02.022 PMC6783343 30979435

[68] Nazarian A Yashin AI Kulminski AM . Genome-wide analysis of genetic predisposition to Alzheimer's disease and related sex disparities. Alzheimers Res Ther 2019; 11: 5. 30636644 10.1186/s13195-018-0458-8 PMC6330399

[69] Hao S Wang R Zhang Y , et al. Prediction of Alzheimer's disease-associated genes by integration of GWAS summary data and expression data. Front Genet 2018; 9: 653. 30666269 10.3389/fgene.2018.00653 PMC6330278

[70] Wang H Lo MT Rosenthal SB , et al. Similar genetic architecture of Alzheimer's disease and differential APOE effect between sexes. Front Aging Neurosci 2021; 13: 674318. 34122051 10.3389/fnagi.2021.674318 PMC8194397

[71] Kunkle BW Schmidt M Klein HU , et al. Novel Alzheimer disease risk loci and pathways in African American individuals using the African genome resources panel: a meta-analysis. JAMA Neurol 2021; 78: 102–113. 33074286 10.1001/jamaneurol.2020.3536 PMC7573798

[72] Shigemizu D Asanomi Y Akiyama S , et al. Whole-genome sequencing reveals novel ethnicity-specific rare variants associated with Alzheimer’s disease. Mol Psychiatry 2022; 27: 2554–2562. 35264725 10.1038/s41380-022-01483-0 PMC9135624

[73] Machiela MJ Chanock SJ . LDlink: a web-based application for exploring population-specific haplotype structure and linking correlated alleles of possible functional variants. Bioinformatics 2015; 31: 3555–3557. 26139635 10.1093/bioinformatics/btv402 PMC4626747

[74] Cummings J Zhou Y Lee G , et al. Alzheimer's disease drug development pipeline: 2023. Alzheimers Dement (N Y) 2023; 9: e12385. 10.1002/trc2.12385 PMC10210334 37251912

[75] Morris JC . Is Alzheimer's disease inevitable with age?: lessons from clinicopathologic studies of healthy aging and very mild Alzheimer's disease. J Clin Invest 1999; 104: 1171–1173. 10545515 10.1172/JCI8560 PMC409830

