# Supplementary material for: Navigating the blurred boundary: Neuropathologic changes versus clinical symptoms in Alzheimer’s disease, and its consequences for research in genetics
Source: J Alzheimers Dis. 2025 Feb 16;104(3):611–26. doi: 10.1177/13872877251317543 (PMC12231839; doi:10.1177/13872877251317543)
Supplement: sj-docx-2-alz-10.1177_13872877251317543 - Supplemental material for Navigating the blurred boundary: Neuropathologic changes versus clinical symptoms in Alzheimer’s disease, and its consequences for research in genetics [file sj-docx-2-alz-10.1177_13872877251317543.docx]

**Supplemental Material**

**Navigating the blurred boundary: Neuropathologic changes versus clinical symptoms in Alzheimer’s disease, and its consequences for research in genetics**

**Supplemental Table 1.** Details of all the databases evaluated in this study. See Excel file.

**Supplemental Table 2.** List of publications and genes found as associated with Alzheimer's disease. See Excel file.

**Supplemental Table 3.** AD and AD-free assessment for the total sample composition per GWAs, considering the databases used. See Excel file.

**Supplemental Table 4.** Description of number of samples per study and AD/AD-free assessment methods used for all GWAs analyzed in this review. See Excel file.

**Supplemental Table 5.** Minimum, maximum, median and average odds ratio value per GWAs. When possible, only values at discovery phase were included. See Excel file.

**Supplemental Table 6.** Odds ratio values per GWAs. When possible, only values at discovery phase were included. See Excel file.

**Supplemental Table 7.** List of all SNPs found associated with sporadic AD in the GWAs that shared OR and respective p-values. RS numbers, genes, chromosome and position are described. Normalized position (Nposition) was calculated as 0-100 from the total bp of the respective chromosome. N refers to the total number of samples at the study where the entry was found. OR refers to odds ratio. For each SNP is indicated if it was observed as statistically associated in more than one study. Repeat identifies the SNPs that were observed in more than one study. See Excel file.

**Supplemental Figure 1.** (A) Minimum and maximum odds ratio values per GWAs. *Odds ratio related to *APOE* markers or markers in linkage disequilibrium with *APOE*, **Studies using controls with minimum age > 70 years and ***Studies that analyzed AD in African American populations. Red dashed line represents OR=1. (B) Mean and median odds ratio values plotted against the total number of samples. Black dashed lines represent an interval of 4,000-125,000 samples, red dashed line represents OR=1.

**Supplemental Figure 2.** LDmatrix plots for all chromosomes where AD associated SNPs were found in the 31 GWAs that reported OR values. Upper triangle matrix squares gradient colors represents increasing D’ values and downer triangle matrix squares represent increasing R^2^, calculations were performed considering European populations in the LDlink database. Chromosomes 22 and 23, as well as 4 and X were excluded from the analyses since either none or a single marker (in a single study), respectively, were found as AD-associated.  For simplicity sake, data from chromosome 12 were excluded as only 2 distant SNPs were identified as AD-related (each in a single GWAs).

**Supplemental Figure 3.** Marker specific odds ratio per total number of samples for the chromosomes where only independent SNPs were observed as AD-associated. Point shapes consider if the SNP was found in more than one GWAs (repeat, triangle), or otherwise (circle).

**Supplemental Figure 4.** Marker specific odds ratios per total number of samples for all the variants found to be AD-associated in at least one of the analyzed GWAs. LD groups were established considering LDmatrix output (Supplemental Figure 2) and two variants are represented by the same color if and only if they belong to the same LD group. Point shapes consider if the SNP was found in more than one GWAs (repeat, triangle) or otherwise (circle).
